# Exploring Conscious Sedation in Pediatric Oral Surgery: A Non-Randomized Clinical Trial on Safety and Efficacy

**DOI:** 10.3390/children12050604

**Published:** 2025-05-05

**Authors:** Niccolò Cenzato, Lorenzo Pasquali, Gregorio Menozzi, Cinzia Maspero

**Affiliations:** 1 Department of Biomedical, Surgical and Dental Sciences, School of Dentistry, University of Milan, 20122 Milan, Italygregorio.menozzi@unimi.it (G.M.);; 2 Fondazione IRCCS Cà Granda Ospedale Maggiore Policlinico, Maxillo-Facial Surgery and Dental Unit, Via Sforza 35, 20122 Milan, Italy

**Keywords:** sedation, pediatric dentistry, oral surgery, conscious sedation, vital signs

## Abstract

Background: Conscious sedation is a well-established technique used in pediatric dentistry that reduces perioperative stress and pain while maintaining verbal contact with the patient. It is particularly beneficial for anxious, very young, or disabled children, improving cooperation and ensuring airway safety. The objective of this study is to assess the safety and efficacy of conscious sedation in pediatric oral surgery by analyzing vital parameters before and after treatment. Methods: A total of 57 pediatric patients [29 females; 28 males], aged 5–14 years [mean: 9.4], were treated at the Fondazione IRCCS Cà Granda Ospedale Maggiore Policlinico of Milan between September 2022 and June 2024. The patients were divided into two groups: nitrous oxide sedation [Group A] and benzodiazepine sedation [Group B]. Informant consent, medical and dental history, vital signs, and anxiety levels were recorded. A statistical analysis was performed using the Wilcoxon test. Due to the non-randomized design of this study, potential selection bias and limitations in internal validity must be acknowledged. Results: The post-sedation diastolic pressure was significantly higher [*p* = 0.02] in Group b, while other vital parameters remained stable. Anxiety significantly decreased in both groups [*p* < 0.01], with a greater reduction in Group A. Conclusions: Conscious sedation is a safe and effective approach, maintaining stable vital parameters. The increase in diastolic pressure in Group B may be explained by preexisting anxiety and benzodiazepine pharmacodynamics. However, the absence of randomization may have influenced group allocation and outcome interpretation. Given the small sample size and the wide age range of the participants, further studies with larger and more homogeneous populations are necessary to refine and validate pediatric sedation protocols.

## 1. Introduction

Controlling stress and pain during dental treatments is one of the main challenges in pediatric dentistry [1,2,3,4,5]. Growing children often lack the adaptive and cognitive skills needed to cope with the dental environment, making it difficult to provide high-quality care. Psychological approaches alone may not be sufficient to reduce levels of anxiety and panic, potentially jeopardizing the success of dental procedures [6,7,8].

In 1982, the American Dental Association sponsored a conference entitled “The Patient-Dentist Relationship and the Management of Fear, Anxiety and Pain”, where the consensus was unanimous: fear and pain contribute to the avoidance of dental care, with up to 9% of patients not seeking dental treatment at least once a year and up to 21% avoiding it even when necessary [9].

Anxiety regarding dental treatment negatively impacts both the child’s comfort level and the outcome of the procedure. Anxious children tend to anticipate dental treatments unrealistically, leading to uncooperative behavior that makes procedures more difficult to perform, especially in children under 6 years of age, due to behavioral immaturity, anxiety, and fear [10,11,12,13,14].

The prevalence of dental anxiety has been reported to be between 10 and 20% in infants and children [15]. Given these negative implications, early identification of anxious children and associated factors is crucial to guide treatment planning. Dental anxiety in children can be assessed by observing behavior; using psychometric questionnaires completed by parents/guardians or the child; and analyzing physiological indicators, such as altered salivary cortisol levels.

Untreated dental caries may cause pain, interfere with sleep and feeding, and ultimately affect growth and development. At the same time, a negative dental experience can cause long-term psychological harm. In such cases, extractions often become the most practical solution for compromised teeth.

To manage these challenging scenarios, conscious sedation has been proposed [15,16]. Conscious sedation involves the use of one or more drugs to induce a controlled depression of the central nervous system (CNS), allowing dental treatment to proceed while maintaining verbal contact with the patient. This facilitates better control of perioperative stress and pain and enables careful monitoring of vital signs.

During sedation, the patient remains responsive to verbal commands, alone or accompanied by light tactile stimulation. Airway patency is maintained without intervention, and spontaneous ventilation remains adequate. This method is considered safe, practical, and effective for very young children, fearful patients with low pain thresholds, and those with intellectual disabilities. Conscious sedation refers to a pharmacologically controlled state of reduced consciousness, with a wide safety margin that minimizes the risk of unintended loss of consciousness.

Proper pre-sedation evaluation is essential, including airway assessment, fasting status, and understanding of drug pharmacodynamics and pharmacokinetics. The availability of emergency airway equipment, venous access, continuous monitoring, and trained personnel in the recovery area must be ensured [17]. Throughout the procedure, the patient retains protective reflexes, maintains spontaneous breathing, and can respond to verbal and physical stimuli. This makes conscious sedation particularly useful in reducing anxiety, discomfort, and resistance to treatment in uncooperative children [18,19,20].

The goal is to achieve an optimal level of sedation that reduces pain and anxiety, enhances amnesia, ensures behavioral control, and maintains stable cardiovascular and respiratory function. Sedative drugs may be administered via various routes—oral, inhalation, nasal, intramuscular, subcutaneous, or intravenous—provided that rigorous safety protocols are followed to minimize risks of respiratory depression and ensure sufficient treatment time [21,22].

Sedation is also commonly used as a premedication before general anesthesia to relax patients while preserving cardiopulmonary reflexes [23,24].

This study aims to evaluate whether conscious sedation is an effective, safe, and controllable procedure by analyzing vital parameter values before and after sedation to verify that they remain within clinically acceptable ranges.

## 2. Materials and Methods

A sample of 57 pediatric patients was selected from the Conscious Sedation Service of the UOC Maxillofacial Surgery and Dentistry Unit at Fondazione IRCCS Ca’ Granda Ospedale Maggiore Policlinico of Milan. The sample comprised 29 females and 28 males aged between 5 and 14 years, with a mean age of 9.4 years.

All the patients were recruited between September 2022 and June 2024. Specific informed consent forms for study enrollment were provided to the parents of the patients.

The patients were assigned to Group A (basic sedation) or Group B (advanced sedation) following an evaluation that considered several factors, which will be detailed subsequently, including the patient’s level of anxiety, cooperation ability, the presence of severe anxiety or dental phobia, and behavioral disorders that could compromise the successful outcome of the intervention.

The inclusion criteria were patients aged ≤14 years, of both sexes, requiring oral surgery, who were uncooperative due to young age or severe anxiety and dental phobia, either healthy or with varying degrees of disability affecting their cooperation, and who were already being treated at the hospital or had previously failed treatment at outpatient facilities and were referred specifically to the Conscious Sedation Unit.

The exclusion criteria were patients aged >14 years or those who underwent the intervention using iatrosedation (non-pharmacological sedation techniques).

A full medical history was taken during the first visit to find out how anxious the patient was using the Venham test [25,26]. The level of cooperation of the patient was also noted, along with any conditions like severe anxiety, dental phobia, or behavioral disorders that might make intervention less effective. Based on the patient’s overall assessment and their Venham test score, the sedation team determined the appropriate approach and assigned the patient to the corresponding study group.

Group 0 (not included in this study): This group was only given behavioral techniques (iatrosedation), which could make the procedure possible without the use of drugs.

Group A: The patients in this group underwent a first-line approach to sedation using basic sedation if iatrosedation was insufficient. Basic sedation involved titration of nitrous oxide, starting at a concentration of 30% and gradually increasing until the desired level of cooperation was achieved.

Group B: The patients who, after an initial evaluation, were not considered suitable for treatment as part of Group A were included in this group. If the use of nitrous oxide was deemed ineffective, an additional visit was scheduled. This included the presence of a second dental sedation specialist and an anesthesiologist to evaluate the use of advanced conscious sedation, moving beyond basic sedation.

For both basic and advanced sedation, a medical examination was conducted. This included obtaining a medical history, a physical examination with the recording of vital signs (systolic pressure, diastolic pressure, heart rate, and SpO_2_), and assessing anxiety levels using the Venham test. All the parameters were recorded before and after the intervention.

The patients were informed about preoperative dietary restrictions. Information about the pre- and postoperative periods was provided in written form via a consent document completed by the dentist and delivered directly to the adult patient, parent, or legal guardian of the minor. This document had to be signed by the patient or their guardian in the case of individuals unable to consent or by both parents in the case of minors.

Basic sedation was performed using nitrous oxide through inhalation sedation, while advanced sedation involved the combined use of inhaled nitrous oxide and sedative benzodiazepines, such as midazolam, administered intravenously. The patients underwent oral surgery procedures, including tooth extractions (4; 9.3%), supernumerary tooth extractions (10; 23.3%), root residual extractions (23; 53.5%), germectomies (5; 11.6%), and lesion removals (1; 2.3%).

A descriptive and inferential statistical analysis was conducted on the collected data. The parameters assessed in this study were systolic blood pressure, diastolic blood pressure, heart rate, oxygen saturation, and anxiety.

The sample was described, with the discrete variables expressed as absolute frequencies and relative percentages and the continuous variables expressed as means (SD). The Spearman correlations were calculated between clinical parameters pre- and post-sedation. The clinical parameters (systolic blood pressure, diastolic blood pressure, heart rate, and oxygen saturation) and anxiety levels before and after sedation in the two groups were tested using the Wilcoxon signed-rank test for paired data. The Wilcoxon rank-sum test (Mann–Whitney U test) was selected due to the non-normal distribution of the data, making it suitable for comparing independent groups (nitrous oxide vs. benzodiazepine sedation).

The distributions of the same parameters were compared between the two sedation groups pre- and post-treatment using the Wilcoxon Mann–Whitney test for independent data. The significance level was set at α = 0.05.

All the analyses were performed using STATA 18 (StataCorp. 2023. Stata Statistical Software: Release 18. College Station, TX, USA, StataCorp LLC).

## 3. Results

Table 1 presents the distribution of the main characteristics of the group and the distribution of the procedures.

The mean age was approximately 9 years. The gender distribution was nearly equal. Advanced sedation was administered more frequently, at a 4:1 ratio. The most common procedure was the extraction of root residuals, performed on 23 of 43 children (53%).

**Figure 1:**
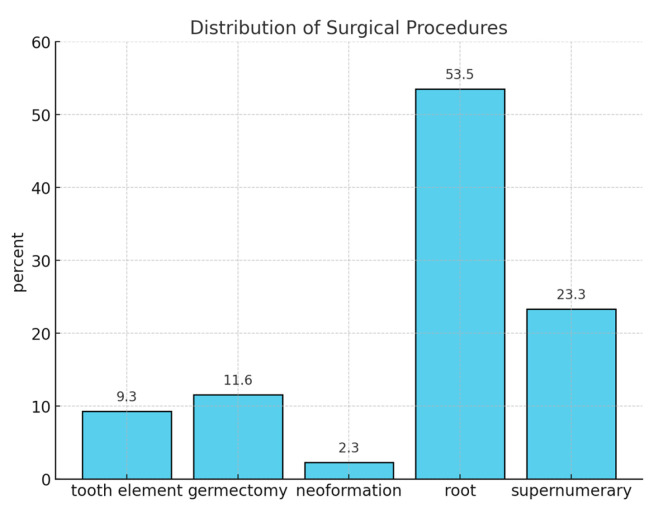
Distribution of surgical procedures.

Figure 1 shows the most frequent procedures, starting from the extraction of root residuals (23, 53.5%), supernumerary teeth (10, 23.3%), and germectomies (5, 11.6%).

**Figure 2:**
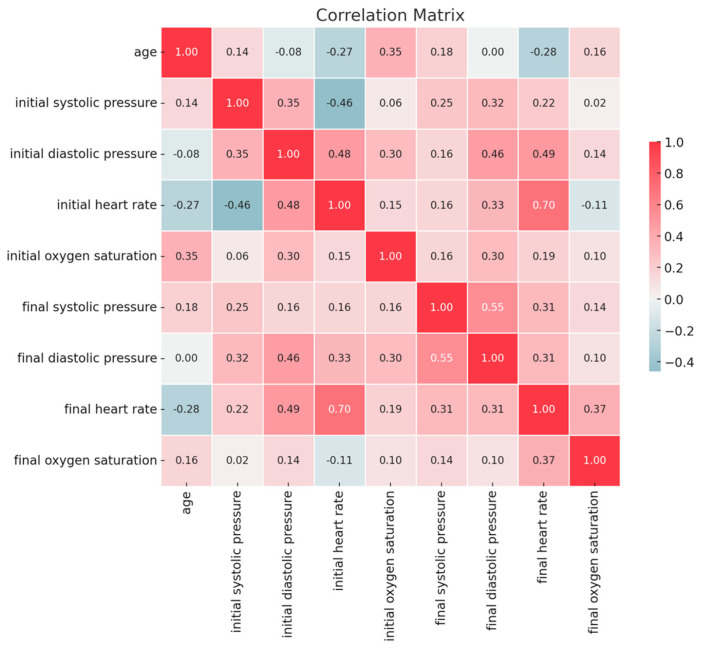
Correlations between clinical parameters pre- and post-sedation.

As appears in Figure 2, the initial heart rate shows a moderate-to-high positive correlation with the final heart rate (r = 0.70; *p* < 0.01).

Initial diastolic pressure shows a moderate positive correlation with final diastolic pressure (r = 0.46; *p* < 0.01).

Initial systolic pressure shows a moderate correlation with initial heart rate (r = 0.46; *p* < 0.01). Initial diastolic pressure shows a moderate correlation with initial heart rate (r = 0.48; *p* < 0.01) and final heart rate (r = 0.49; *p* < 0.01).

Final systolic pressure shows a moderate correlation with final diastolic pressure (r = 0.55; *p* < 0.01).

As shown in Table 2, none of the clinical parameters showed significant differences. However, the Venham test score decreased from 6.7 (0.5) to 4.4 (1.0); *p* = 0.01.

In the advanced sedation group, none of the clinical parameters showed significant differences. However, the Venham test score decreased from 6.9 (0.3) to 4.9 (0.3); *p* < 0.01.

Pre-sedation, there were no differences between the observed clinical parameters.

However, the Venham test score was higher in the advanced sedation group: 6.9 (0.5) vs. 6.7 (0.3); *p* < 0.01.

In Table 3, post-sedation diastolic pressure appeared to increase in the patients with advanced sedation: 68.2 (12.4) vs. 59.1 (6.1) mmHg; *p* = 0.02.

No differences were observed in the other clinical parameters.

Regarding the Venham test score, the patients with advanced sedation had a higher value: 4.9 (0.3) vs. 4.4 (1.0); *p* < 0.01.

**Figure 3:**
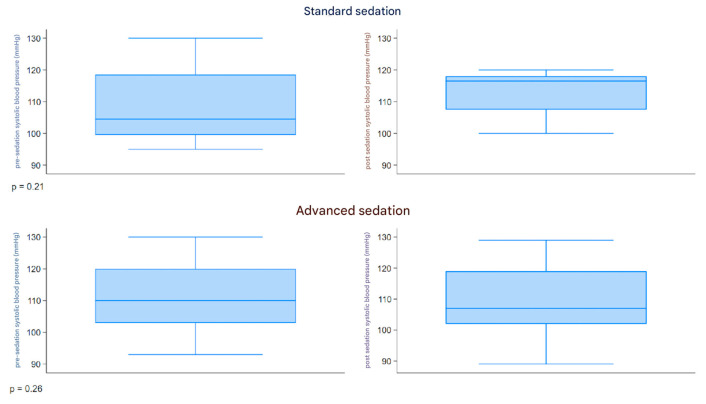
Boxplot systolic pressure.

The boxplot described in Figure 3 represents the distribution of the systolic pressure (mmHg) measured before and after sedation, comparing the two groups. Both groups had a similar range of 90–130 mmHg pre-sedation and 89–129 mmHg post-sedation. The pre- and post-sedation values were similar in both groups.

**Figure 4:**
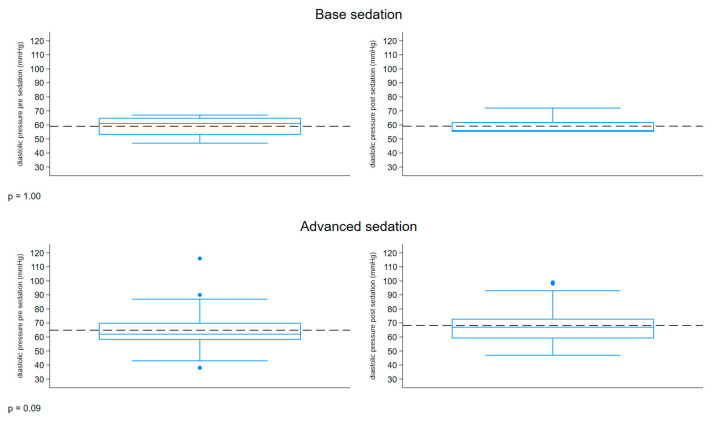
Boxplot diastolic pressure.

The boxplot described in Figure 4 represents the distribution of the diastolic pressure (mmHg) measured before and after sedation, comparing the two groups. Both groups had a similar range of 38–116 mmHg pre-sedation and 47–99 mmHg post-sedation. An increase in diastolic pressure was observed in the patients with advanced sedation post-treatment.

**Figure 5:**
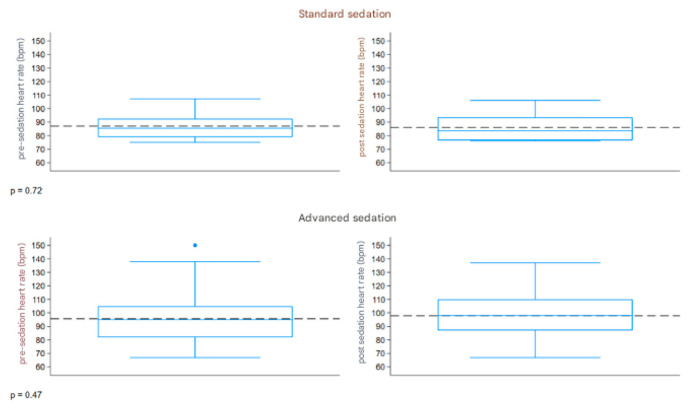
Boxplot heart rate. The dashed line refers to the reference values.

The boxplot described in Figure 5 represents the distribution of the heart rate (bpm) measured before and after sedation, comparing the two groups. Both groups had a similar range of 67–150 bpm pre-sedation and 67–137 bpm post-sedation. The pre- and post-sedation values were similar in both groups.

**Figure 6:**
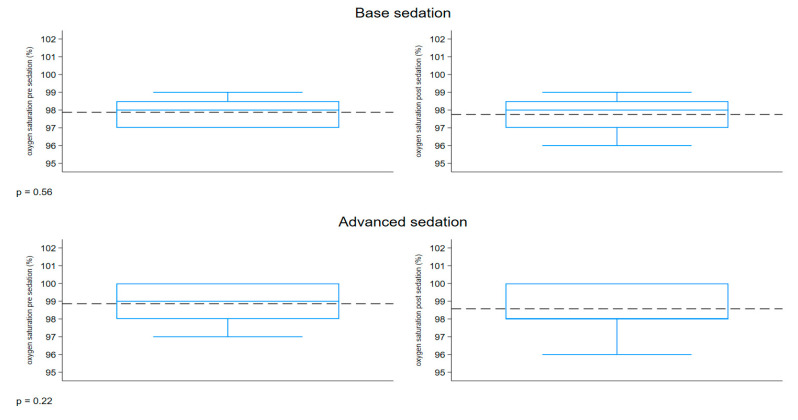
Boxplot oxygen saturation.

The boxplot described in Figure 6 represents the distribution of the oxygen saturation (%) measured before and after sedation, comparing the two groups. Both groups had a similar range of 97–100% pre-sedation and 96–100% post-sedation. The pre- and post-sedation values were similar in both groups.

## 4. Discussion

The results confirmed strong and significant correlations between certain physiological parameters, particularly between systolic and diastolic blood pressure post-sedation, as well as between pre- and post-sedation heart rate values. As demonstrated in the literature, this study found a statistically significant difference in systolic and diastolic blood pressure among patients treated with benzodiazepines, as did the study by Wang et al. [20,27,28]. Similarly, the study by Behrman et al. [29] noted that a change in systolic pressure of 40–50 mmHg due to excitement, crying, or physical responsiveness is considered physiological in pediatric patients. Therefore, while the changes in mmHg observed in the following studies are significant, they are not clinically relevant. Regarding heart rate, Behrman et al. [29] emphasized its variability throughout the day, which explains the absence of fixed baseline values but rather a range defining an interval. This range also depends on the patient’s age, as preschool-aged children can exhibit fluctuations of 40–50 beats per minute. The results obtained in this study can be compared with other findings, such as those of Tavassoli-Hojjati et al. [30], where heart rate post-midazolam administration remained within the physiological range. Analyzing the data reveals that clinical parameters remained constant over time in both groups, with no significant variations observed between pre- and post-sedation.

This finding is noteworthy, as it appears to confirm that there is no adverse impact on patients undergoing advanced sedation, making it as safe as basic sedation. The same conclusion was drawn by Vasakova et al. [27] who, in a study of 272 pediatric patients, highlighted the effects of midazolam on vital signs. Their findings support the idea that while there may be an increase in blood pressure and a decrease in oxygen saturation, these values remained within physiological limits [31]. Additionally, older children tended to exhibit better behavior during sedation, highlighting the influence of age on sedation outcomes [27]. Similarly, Mourad et al. [32,33], in a retrospective study evaluating the success of N_2_O sedation for pediatric dental treatment, emphasized its high success rates and potential as an effective therapeutic option for preoperative and anxious children. This study also noted that oxygen saturation remained above 95% throughout the procedure, corroborating findings from an observational clinical study by Lourenço-Matharu and Roberts [34], which used the same dose of midazolam.

When comparing the two groups on post-sedation clinical parameters, an increase in diastolic blood pressure was observed in patients undergoing advanced sedation, despite similar pre-sedation values. This difference can be attributed to several factors. First, patients undergoing advanced sedation scored higher on the Venham scale compared to those undergoing basic sedation. It is reasonable to assume that these children were more agitated due to various conditions, including systemic diseases, behavioral disorders, negative past dental experiences, age, administered dose, and body constitution. Furthermore, as Wang et al. [28] explained, blood pressure is currently defined as a function of two continuous variables: age and height for each sex. This study demonstrated that systolic and diastolic blood pressure increased with age in pre-sedation measurements, while no statistically significant differences were observed post-sedation, as values remained within a clinically acceptable range. Additionally, there is a strong correlation between blood pressure changes and the administered dose, which corresponds to the patient’s weight, age, and height. Wang et al. [28] highlighted that higher doses of midazolam may result in higher systolic and diastolic pressures, as well as lower heart rates. Similarly, Lenahan et al. [35] focused on factors influencing the effectiveness of sedation, such as patient age, pre-sedation behavior, and willingness to take medication. The primary goal of Lenahan et al.’s study was to evaluate the safety and efficacy of combining meperidine and hydroxyzine for oral sedation in anxious or uncooperative pediatric patients. Secondary objectives included identifying potential factors impacting sedation effectiveness. The study reviewed 248 electronic medical records of pediatric patients [131 females; 117 males] who received meperidine and hydroxyzine sedation. The results showed that this combination was safe and effective, with over 81% of sedations deemed effective or highly effective. No severe side effects were reported, and only 5% of sedations were interrupted due to patient behavior. This study demonstrates that meperidine and hydroxyzine are a viable option for pediatric sedation during dental treatments [35]. Regarding blood pressure variations, Wan et al. [36] compared patients treated with midazolam to those treated with a placebo, demonstrating that diastolic pressure in the midazolam group did not significantly differ from the control group. This suggests that benzodiazepine use is both effective and safe. Anxiety assessment showed that both groups significantly reduced their scores by at least two points. However, patients undergoing basic treatment showed a greater reduction in anxiety, likely due to their baseline Venham scores being lower by two-tenths, indicating less initial anxiety. Nonetheless, children undergoing deep sedation also exhibited a significant reduction in their scores, confirming the treatment’s efficacy. Antunes et al. [37] discussed the long-term effects of pharmacological management on children’s behavior during dental treatment. They found that moderate sedation with midazolam or midazolam/ketamine improved behavior in subsequent dental sessions compared to those without sedation. This underscores the importance of effective pharmacological management in reducing the risk of adverse dental experiences, particularly when treating early-life caries [37]. A recent systematic review reported that anxiety in preschool-aged children was primarily assessed using proxy measures (mainly parent reports) in most studies published since 1998 [15]. Children six years and older were the only ones who could do self-evaluations [15]. This was true even though there were validated tools for preschoolers, like the Facial Image Scale [38] and the Venham Picture Test [39]. It is also recommended to inform parents about expected post-sedation effects based on the administered sedation regimen. It was noted that parents of patients sedated with specific drug combinations were more concerned about their child’s recovery state than those sedated with other combinations, suggesting that parental reactions vary depending on sedation effects. Proper primary and secondary prevention of major pathologies helps avoid more severe clinical situations [40]. Good oral health practices from an early age are essential for ensuring long-term positive dental health and hygiene outcomes [41] as well as to check the patients regularly to motivate them to be careful about oral hygiene [42], especially in injured primary teeth where careful checkups must be programmed to supervise the permanent tooth eruption.

Finally, it can be concluded that there is no universally recognized basic sedation regimen for such procedures, and each agent or combination may yield varying results and side effects [43,44,45].

This study is an experimental clinical trial, with a limited and unbalanced sample [base-to-advanced sedation ratio of 1:4] due to the difficulty of interventions and behavioral disorders in patients requiring deeper sedation. Approximately 25% of patients were not treated due to intraoperative issues, such as poor responsiveness to sedation or uncontrolled anxiety. The Venham score presented many missing data points for patients with physical disabilities, as verbal communication was not possible. Moreover, the Venham test is more suitable for children aged 6–10, given their greater emotional awareness at this age.

## 5. Conclusions

Inhalation conscious sedation is commonly used in pediatric dentistry, proving to be a safe technique with good results. The vital parameters of BP, HR, and SpO_2_ measured at the end of the procedure were clinically acceptable; however, a further controlled and randomized clinical trial is needed to confirm the pharmacodynamic relationship given that this can be considered a pilot study. The results obtained in this study, as assessed by the Venham test, further support the efficacy of both basic and advanced sedation, with reduced post-procedure scores recorded.

## Figures and Tables

**Table 1:** General characteristics of the patients.

Variable	*N*	%
**Total**	43	100
**Age, mean [DS]**	9.4 [2.2]	
**Gender**		
Male	20	47
Female	23	53
**Type of sedation**		
Basic	8	19
Advanced	35	81
**Benzodiazepine dose, mean [DS]**	10.8 [4.5]	
**Type of surgery**		
Root residual	23	53
Supernumerary teeth	10	23
Germectomy	5	12
Tooth extraction	4	9
Lesion removal	1	2

**Table 2:** Comparison of clinical parameters and Venham scores pre- and post-sedation.

Variable	N [%]	Pre-Sedation Mean [DS]	Post-Sedation Mean [DS]	*p*-Value ^a^
**Base Sedation**	8 (9)			
**Clinical Parameter**				
Systolic pressure [mmHg]		108.8 [12.2]	113 [7.2]	0.21
Diastolic pressure [mmHg]		59 [7.4]	59.1 [6.1]	1.00
Heart rate [bpm]		87 [10.6]	86.1 [11.1]	0.72
Oxygen saturation [%]		97.9 [0.8]	97.8 [1.0]	0.56
**Venham score**		6.7 [0.5]	4.4 [1.0]	0.01
**Advanced Sedation**	35 (81)			
**Clinical Parameter**				
Systolic pressure [mmHg]		111.8 [10.9]	108.7 [10.7]	0.26
Diastolic pressure [mmHg]		64.8 [14.5]	68.2 [12.4]	0.09
Heart rate [bpm]		95.6 [18.9]	97.8 [11.2]	0.47
Oxygen saturation [%]		98.9 [1.00]	98.6 [1.1]	0.22
**Venham score**		6.9 [0.3]	4.9 [0.3]	<0.01

^a^ calculated with the Wilcoxon paired test.

**Table 3:** Comparison of clinical parameters and Venham scores between the two sedation groups pre- and post-treatment.

Variable	N [%]	Advanced Mean [DS]	N [%]	Base Mean [DS]	*p*-Value ^a^
**Pre-sedation**	35 [81]		8 [19]		
**Clinical Parameter**					
Systolic pressure [mmHg]		111.8 [10.9]		108.8 [12.2]	0.43
Diastolic pressure [mmHg]		64.8 [15.4]		59 [7.4]	0.36
Heart rate [bpm]		95.6 [18.9]		87 [10.6]	0.21
Oxygen saturation [%]		98.9 [1.0]		97.9 [0.8]	**0.02**
**Venham score**	9 [56]	6.9 [0.3]	7 [44]	6.7 [0.5]	**0.01**
**Post-sedation**	35 [81]		8 [19]		
**Clinical Parameter**					
Systolic pressure [mmHg]		108.7 [10.7]		113 [7.2]	0.33
Diastolic pressure [mmHg]		68.2 [12.4]		59.1 [6.1]	**0.02**
Heart rate [bpm]		97.8 [11.2]		86.1 [11.1]	0.06
Oxygen saturation [%]		98.6 [1.1]		97.8 [1.0]	0.07
**Venham score**	9 [56]	4.9 [0.3]	7 [44]	4.4 [1.0]	**<0.01**

^a^ calculated with the Wilcoxon [Mann–Whitney] test.

## Data Availability

The data presented in this study are available upon reasonable request, after the signature of a formal data-sharing agreement in an anonymous form, from the corresponding author because they are protected by privacy.
